# Synthesis, crystal structure and Hirshfeld surface analysis of (*Z*)-2-({amino­[6-phenyl-3-(4-methylphen­yl)-1,2,4-triazin-5-yl]methyl­idene}amino)-6-meth­oxy­benzo[*d*]thia­zole-4,7-diyl di­acetate

**DOI:** 10.1107/S2056989026008972

**Published:** 2026-09-08

**Authors:** Yaroslav K. Shtaitz, Dmitry S. Kopchuk, Vasiliy S. Gaviko, Menberu Mengesha Woldemariam, Mehmet Akkurt, Nizami A. Ekberov, Asmet N. Azizova

**Affiliations:** a The Ural Federal University named after the first president of Russia B N Yeltsin Mira 19 st 620002 Yekaterinburg Russian Federation; b Postovsky Institute of Organic Synthesis, Ural Branch of the Russian Academy of Sciences, Sof’i Kovalevskoy 22 st., 620137, Yekaterinburg, Russian Federation; c Sirius University of Science and Technology, Olympic Avenue 1, 354340, Sirius Federal Territory, Russian Federation; d M N Mikheev Institute of Metal Physics of the Ural Branch of the Russian Academy of Sciences Sof'i Kovalevskoy 18 st 620108 Yekaterinburg Russian Federation; e Department of Physics, Jimma University, Jimma, Ethiopia; f Department of Physics, Faculty of Sciences, Erciyes University, 38039 Kayseri, Türkiye; g Azerbaijan State Pedagogical University, 68 Uzeyir Hajibeyov St., AZ 1000, Baku, Azerbaijan; h Scientific Research Centre (SRC), Azerbaijan Medical University, A. Kasumzade, St. 14, AZ 1022, Baku, Azerbaijan; i Scientific Research Center, Baku Engineering University, Hasan Aliyev str. 120, AZ0101, Khirdalan, Absheron, Azerbaijan; Universidade Federal do ABC, Brazil

**Keywords:** crystal structure, 1,3-benzo­thia­zole, 1,2,4-triazine, hydrogen bonds, disorder, Hirshfeld surface analysis

## Abstract

The stable conformation of the title mol­ecule, C_29_H_24_N_6_O_5_S, is caused by two intra­molecular N—H⋯N hydrogen bonds producing fused *S*(5) and *S*(6) rings. In the crystal, mol­ecules are connected by inter­molecular N—H⋯O inter­actions along the *b*-axis direction, forming *C*(9) chains. Furthermore, there are π–π inter­actions [centroid–to-centroid distance = 3.8200 (12) Å] between the benzene ring of the *p*-tolyl group and the 1,2,4-triazine ring in the same direction. As a result, the mol­ecules form ribbons along the *b*-axis direction,. A Hirshfeld surface analysis was performed, showing the predominance of H⋯H, O⋯H/H⋯O, C⋯H/H⋯C and N⋯H/H⋯N contacts.

## Abstract 2

In the crystal, mol­ecules are connected by inter­molecular N—H⋯O inter­actions and π–π inter­actions [centroid–to-centroid distance = 3.8200 (12) Å], forming ribbons along the *b*-axis direction.

## 1. Chemical context

Metal-mediated or metal-free activation of nitriles (C≡N triple bonds) is a powerful synthetic strategy for the functionalization of organo­nitriles to form new N-heterocycles, ligands, metal complexes, catalysts, *etc*. (Kopylovich *et al.*, 2011 ▸; Mahmudov *et al.*, 2014 ▸). Recently, we developed a new synthetic method for functionalizing the C≡N nitrile group of 5-cyano-1,2,4-triazines with heterocyclic amines via a nucleophilic *ipso*-substitution reaction (Krinochkin *et al.*, 2022 ▸; Rammohan *et al.*, 2021 ▸); therefore, as a continuation of this work, we investigated 2-amino­benzo­thia­zole as a nucleophile. It was found, that upon solvent-free inter­action of 6-phenyl-3-(*p*-tol­yl)-1,2,4-triazine-5-carbo­nitrile (**1**) with 6-meth­oxy­benzo[*d*]thia­zol-2-amine (**2**) at 423 K, the reaction yields the unexpected product (*Z*)-*N*′-(6-meth­oxy­benzo[*d*]thia­zol-2-yl)-6-phenyl-3-(*p*-tol­yl)-1,2,4-triazine-5-carboximidamide (**3**). An attempt of oxidative cyclization of a six-membered intra­molecular resonance-assisted hydrogen-bonding synthon (Mahmudov & Pombeiro, 2016 ▸) in (**3**) with lead tetra­acetate in a benzene–acetic acid mixture (Mishra *et al.*, 2007 ▸) resulted in the di­acyl­ated product (*Z*)-2-({amino­[6-phenyl-3-(*p*-tol­yl)-1,2,4-triazin-5-yl]methyl­ene}amino)-6-meth­oxy­benzo[*d*]thia­zole-4,7-diyl di­acetate (**4**). The structures of the obtained compounds were confirmed by ^1^H NMR spectroscopy, mass spectrometry, and elemental analysis, as well as by single-crystal X-ray diffraction for compound (**4**).
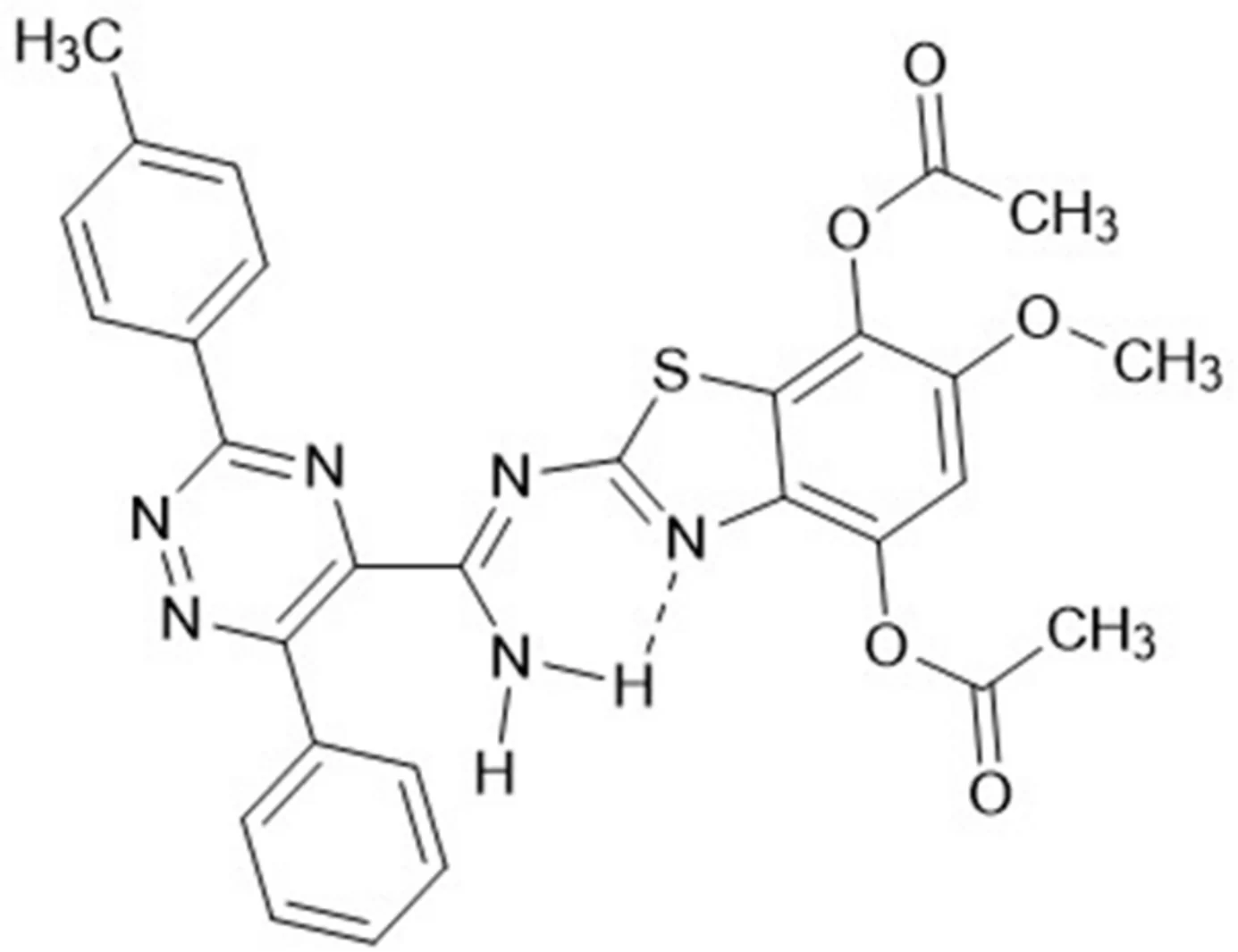


## 2. Structural commentary

**Figure 1:**
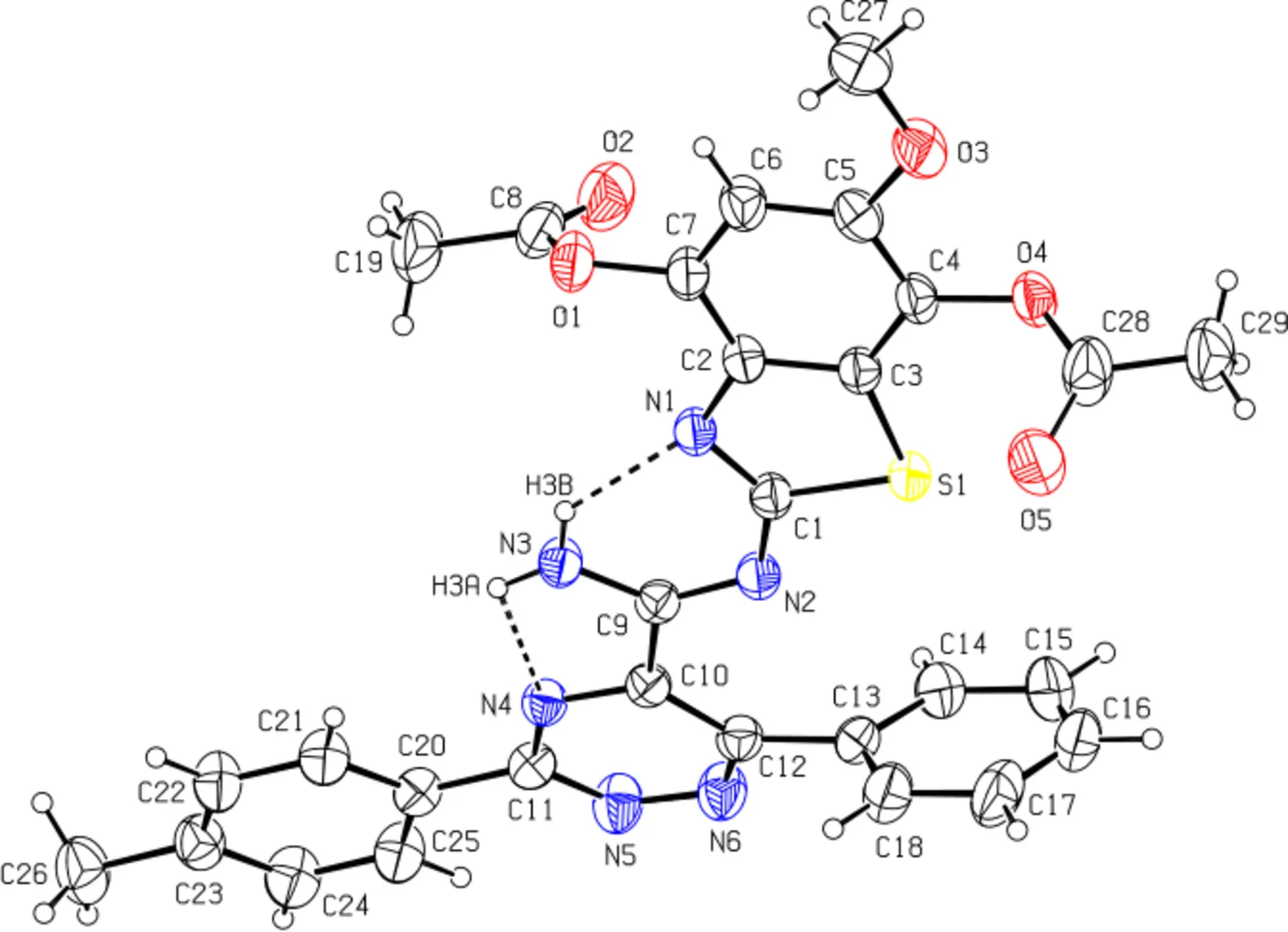
Mol­ecular structure of the title compound showing the atomic labelling. Displacement ellipsoids are drawn at the 30% probability level.

The title mol­ecule (Fig. 1 ▸) has a stable conformation due to two intra­molecular N—H⋯N hydrogen bonds that produce fused *S*(5) and *S*(6) rings (Table 1 ▸; Bernstein *et al.*, 1995 ▸). In a six-membered resonance-assisted hydrogen-bonding (RAHB) ring, the N3—H3*B*⋯N1 bond with [N⋯N distance = 2.676 (2) Å] falls in the N⋯N distance range [2.542–2.897 Å] observed for other supra­molecular synthons (Gurbanov *et al.*, 2020 ▸), and indicates a low delocalization within the heterodienic moiety. The N—H⋯N angle of 130.8 (19)° differs significantly from the average O—H⋯O angle (149°; Bertolasi *et al.*, 1993 ▸) of β-diketone enols involved in similar intra­molecular RAHB.

The 1,3-benzo­thia­zole ring system (S1/N1/C1–C7) is essentially planar with a r.m.s. deviation of 0.0255 Å. The least-squares plane of this ring system forms angles of 17.2 (1), 81.2 (1), and 20.4 (1)° with the ring planes of the 1,2,4-triazine ring (N4–N6/C10–C12) and the benzene ring planes (C13–C18 and C20–C25) of its attached phenyl and 4-methyl­phenyl groups, respectively. The rings (C13–C18 and C20–C25) of the phenyl and 4-methyl­phenyl groups form angles of 62.1 (1)° with each other, while they make angles of 64.0 (1) and 5.8 (1)°, respectively, with the 1,2,4-triazine ring plane (N4–N6/C10–C12).

## 3. Supra­molecular features

**Figure 2:**
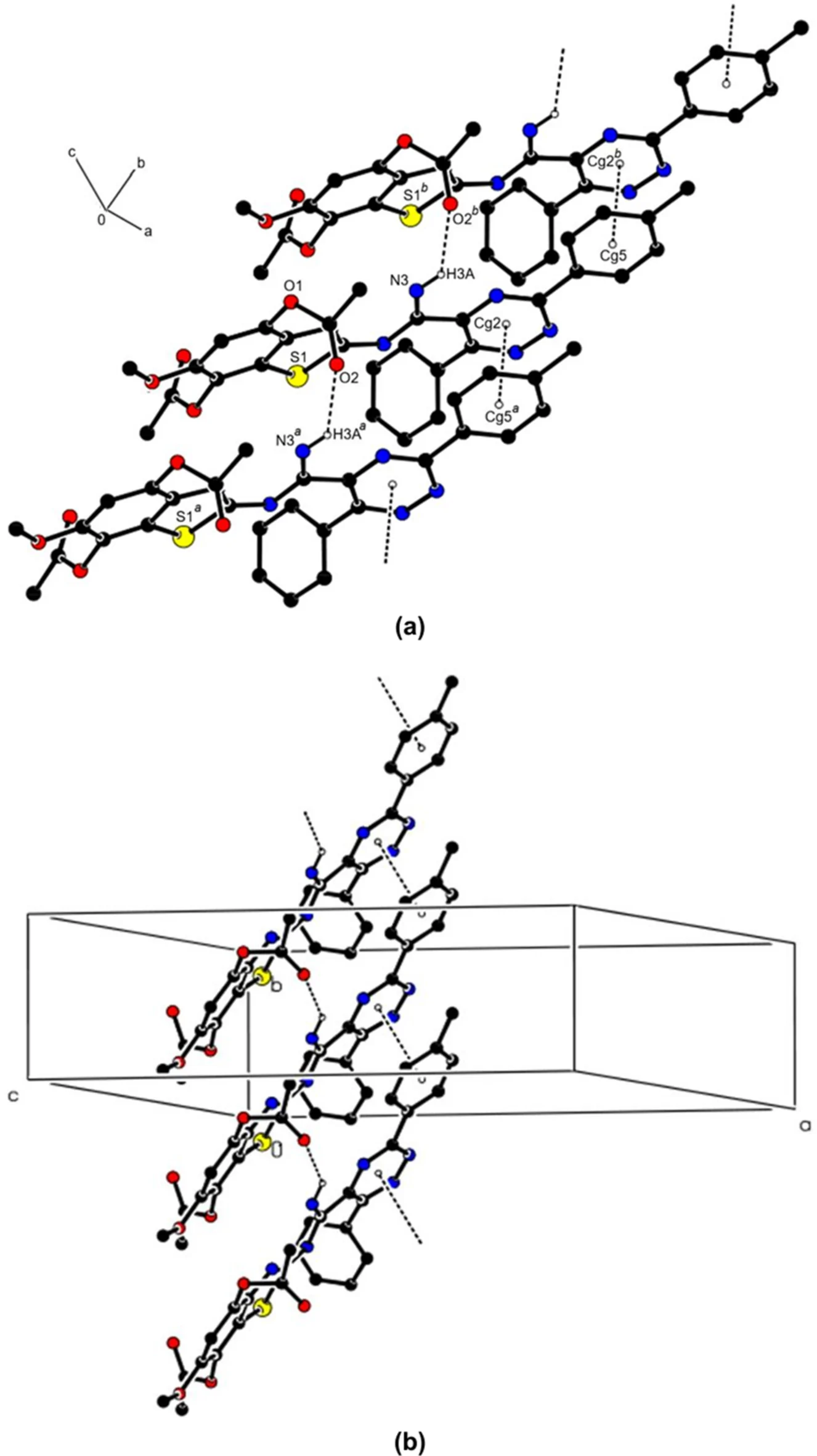
(*a*) Partial view of N—H⋯O hydrogen bonds and π–π inter­actions (dashed lines) in the unit cell of the title compound. H atoms not involved in hydrogen bonding and the minor component of the disordered O5 atom are omitted. (*b*) A different view of these inter­actions within a unit cell.

In the crystal, the mol­ecules, arranged at angles of approximately 45° to the *a*-axis direction and at equal inter­vals, are linked by inter­molecular N—H⋯O inter­actions (Table 1 ▸) along the *b*-axis direction, forming *C*(9) chains. In addition, there are π-π inter­actions [*Cg*2⋯*Cg*5^*a*^ = 3.8200 (12) Å, shift = 1.107 Å and *Cg*5⋯*Cg*2^*b*^ = 3.8200 (12) Å, shift = 0.963 Å; symmetry codes (*a*) *x*, −1 + *y*, *z* and (*b*) x, 1 + *y*, *z*] between the centroids *Cg*2 and *Cg*5 of the 1,2,4-triazine ring and the benzene ring of the *p*-tolyl group, along the *b*-axis direction (Fig. 2 ▸*a*,*b*). Thus, the mol­ecules form ribbons along the *b*-axis direction. The cohesion of the packing is ensured by C—H⋯O inter­actions between these ribbons.

## 4. Hirshfeld surface analysis

**Figure 3:**
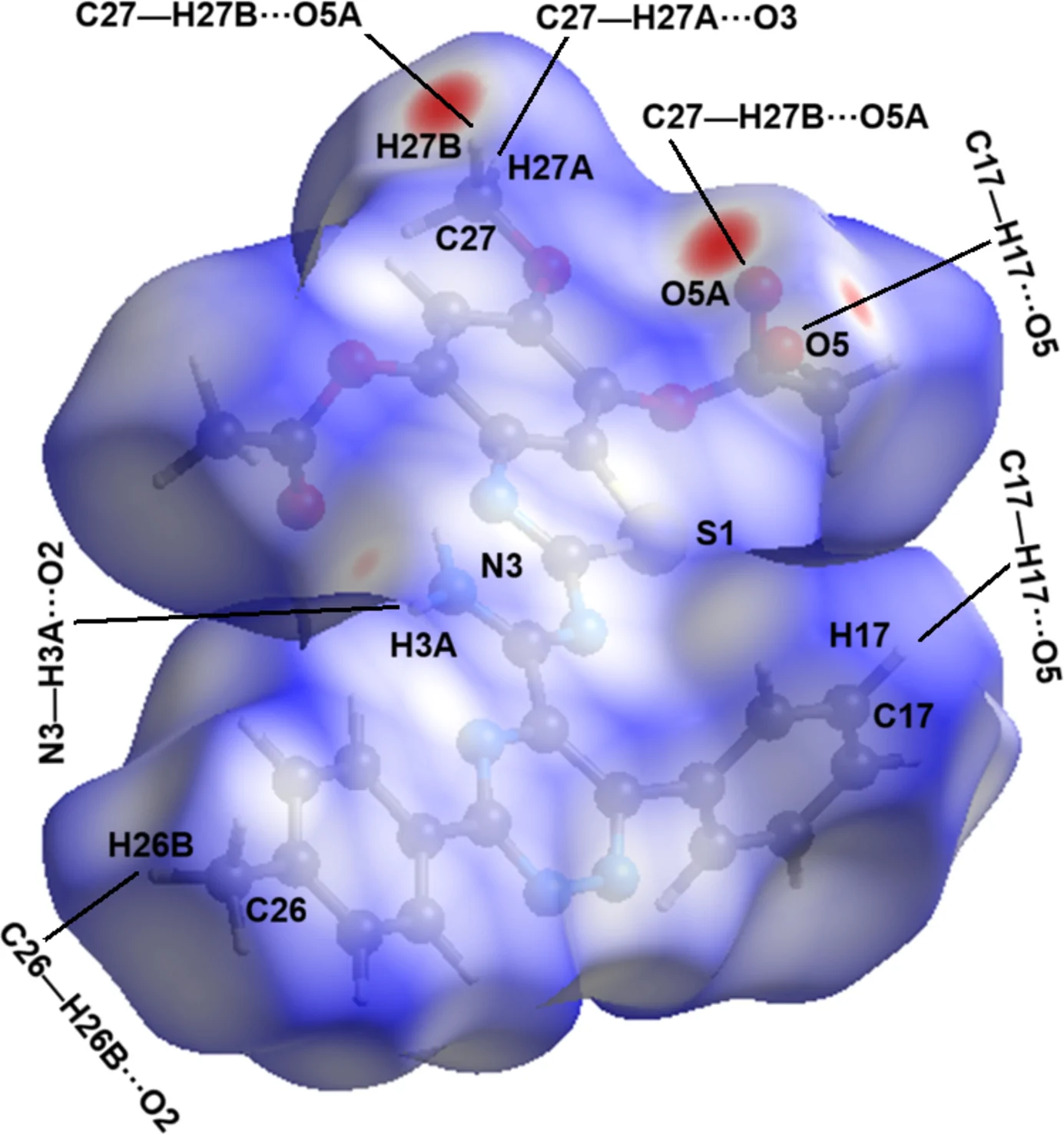
Hirshfeld surface of the title compound mapped over *d*_norm_.

**Figure 4:**
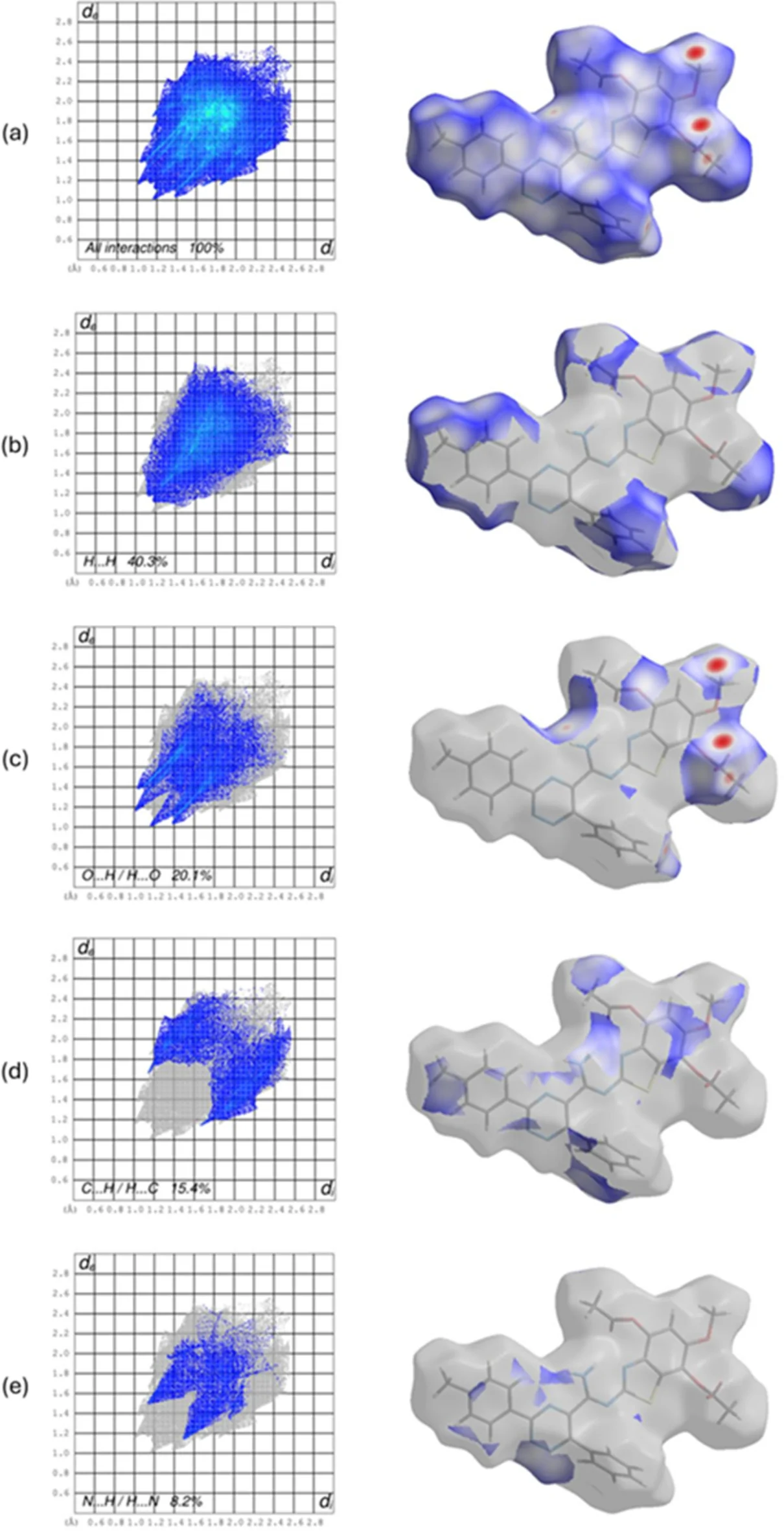
Hirshfeld surface mapped over *d*_norm_ and the corresponding two-dimensional fingerprint plots for the title compound. (*a*) All inter­actions (100%) and the relative contributions of (*b*) H⋯H (40.3%), (*c*) O⋯H/H⋯O (20.1%), (*d*) C⋯H/H⋯C (15.4%) and (*e*) N⋯H/H⋯N (8.2%) inter­actions to the Hirshfeld surface are indicated. The *d*_i_ and *d*_e_ values are the closest inter­nal and external distances (in Å) from given points on the Hirshfeld surface.

A Hirshfeld surface analysis and the corresponding fingerprint plots were generated using *CrystalExplorer* software (Spackman *et al.*, 2021 ▸) to further investigate and qu­antify the contributions of the various inter­molecular inter­actions in the crystal. The Hirshfeld surface mapped over *d*_norm_ using a fixed colour scale of −0.3005 (red) to 1.3598 (blue) a.u. and corresponding colours representing various inter­actions is shown in Fig. 3 ▸. The two-dimensional fingerprint plots for all inter­molecular inter­actions and those delineated into specific contacts are shown in Fig. 4 ▸. The largest contribution comes from H⋯H contacts at 40.3% of the total, which is consistent with the significant hydrogen content of the mol­ecule. The next most important contact is O⋯H/H⋯O at 20.1%, which primarily comes from the intra­molecular O—H⋯O and inter­molecular N—H⋯O as well as C—H⋯O inter­actions. The C⋯H/H⋯C inter­actions account for 15.4% while N⋯H/H⋯N contacts contribute 8.2%, followed by N⋯C/C⋯N contacts contributing 4.0%. Further, 3.9, 2.2, 1.2, 1.1, 0.5, 0.2 and 0.1% contributions corresponding to S⋯H/H⋯S, O⋯C/C⋯O, S⋯C/C⋯S, O⋯N/N⋯O, N⋯N, S⋯O/O⋯S and S⋯N/N⋯S contacts, respectively, are also observed.

## 5. Database survey

A search of the Cambridge Structural Database (CSD, Version 6.00, update of April 2025; Groom *et al.*, 2016 ▸) for the *N*′-(1,3-benzo­thia­zol-2-yl)-1,2,4-triazine-5-carboximidamide unit generated six closely related compounds: (*Z*)-*N*-(4-meth­oxy­benzo[*d*]thia­zol-2-yl)-3,6-di-*p*-tolyl-1,2,4-triazine-5-carb­oximidamide (QARLOP: Shtaitz *et al.*, 2026 ▸), *N*-[(1,3-benzo­thia­zol-2-yl)carbamo­thio­yl]-4-bromo­benzamide (JOM­GOL: Sow *et al.*, 2024 ▸), *N*′-(1,3-benzo­thia­zol-2-yl)benzene­sulfono­hydrazide (COCFOS: Baddeley *et al.*, 2019 ▸), *N*-(1,3-benzo­thia­zol-2-yl)acetamide (TIJPAF: Nayak *et al.*, 2013 ▸), 2-[(6-meth­oxy-1,3-benzo­thia­zol-2-yl)carbonoimido­yl]phenol (SUFFEG: Hijji *et al.*, 2015 ▸) and *N*-(4,6-di­methyl­pyrimidin-2-yl)-1,3-benzo­thia­zol-2-amine (YAJBUI: Mohamed *et al.*, 2011 ▸).

QARLOP crystallizes in the triclinic *P*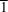 space group while JOMGOL and YAJBUI crystallize in the monoclinic *P*2_1_/*n* space group. COCFOS has two mol­ecules (*A* and *B*) in the asymmetric unit and crystallizes in the ortho­rhom­bic space group *Pbca*. TIJPAF has two mol­ecules (*A* and *B*) in the asymmetric unit and crystallizes in the monoclinic space group *P*2_1_/*c*. SUFFEG has two mol­ecules in the asymmetric unit and crystallizes in the ortho­rhom­bic space group *Pna*2_1_.

In the crystal of QARLOP, inter­molecular C—H⋯O and C—H⋯N hydrogen bonds form layers parallel to the (010) plane. Furthermore, π–π and C—H⋯π [centroid-to-centroid distance = 3.7380 (12) Å] inter­actions connect mol­ecules, forming ribbons along the *a*-axis direction. In JOMGOL, inter­molecular C—H⋯N, C—H⋯S, and N—H⋯S hydrogen bonds form layers parallel to the *ac* plane. In YAJBUI, N—H⋯N hydrogen bonds link the mol­ecules, generating a centrosymmetric cyclic hydrogen-bonded dimer with an 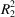
(8) motif. In COCFOS, the *A* and *B* mol­ecules in the asymmetric unit are linked into a supra­molecular dimer via pairwise hydrazinyl-N—H⋯N(thia­zol­yl) hydrogen bonds. Hydrazinyl-N—H⋯O(sulfon­yl) hydrogen bonds between *A* mol­ecules assemble the dimers into chains along the *a*-axis direction, while links between centrosymmetrically related *B* mol­ecules, leading to eight-membered {⋯HNSO}_2_ synthons, link the mol­ecules along [001]. The result is an undulating supra­molecular layer. Layers stack along the *b*-axis direction with benzo­thia­zole-C—H⋯O(sulfon­yl) points of contact being evident. In TIJPAF, the *A* and *B* mol­ecules in the asymmetric unit are linked by N—H⋯N hydrogen-bond pairs, forming 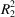
(8) dimer motifs along the [100] axis. In SUFFEG, a pair of C—H⋯O bonds connects two mol­ecules in the asymmetric unit with the ring motif 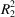
(20). Weak inter­molecular C—H⋯N inter­actions also link these dimers, forming sheets parallel to the *ab* plane.

## 6. Synthesis and crystallization

The starting compound 5-cyano-1,2,4-triazine was prepared according to a literature procedure (Kozhevnikov *et al.*, 2002 ▸), and a known synthetic procedure (Shtaitz *et al.*, 2026 ▸) was used in the synthesis of (**4**).

**Synthesis of compound** (**3**)**.** 0.37 mmol of 6-phenyl-3-(*p*-tol­yl)-1,2,4-triazine-5-carbo­nitrile (**1**) and 0.41 mmol of 6-meth­oxy­benzo[*d*]thia­zol-2-amine (**2**) were added into a round-bottom flask and the resulting mixture was heated at 423 K for 8 h under an inert atmosphere. The product was isolated by column chromatography (eluent AcOEt:CDCl_3_ = 1:9; *R*_f_ 0.7 0.4). The pure product (*Z*)-*N*′-(6-meth­oxy­benzo[*d*]thia­zol-2-yl)-6-phenyl-3-(*p*-tol­yl)-1,2,4-triazine-5-carboximid­amide (**3**) was obtained by recrystallization from ethanol solution. Yield 72 mg (43%). ^1^H NMR (400 MHz, CDCl_3_): *δ* = 2.48 (*s*, 3H, CH_3_), 3.84 (*s*, 3H, OCH_3_), 6.99–7.02 (*dd*, 1H, H-5 (benzo­thia­zole), *J* 8.9 2.6 Hz,), 7.38–7.42 (*m*, 2H, C_6_H_4_CH_3_), 7.45–7.50 (*m*, 3H, Ph), 7.56 (*br. s*, 1H, NH), 7.69–7.72 [*m*, 1H, H-4 (benzo­thia­zole)], 7.77–7.80 (*m*, 2H, Ph), 8.49–8.53 (*m*, C_6_H_4_CH_3_), 10.18 (*br. s*, 1H, NH). Mass-spectrum, *m*/*z* (*I*_rel_, %): m/z: 453.15 [*M* + H]^+^ (100). Found, %: C 66.34, H 4.44, N 18.58. C_25_H_20_N_6_OS. Calculated, %: C 66.35, H 4.45, N 18.57.

**Figure 5:**
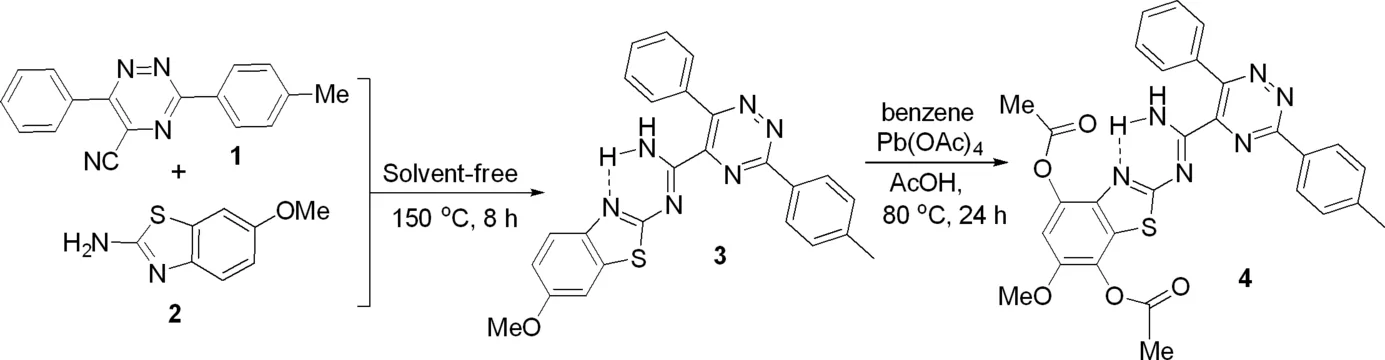
Reaction scheme for the title compound.

**Synthesis of compound** (**4**)**:** To a solution of 203 mg (0.45 mmol) of compound (**3**) in 15 mL of benzene and 5 mL of glacial acetic acid, 399 mg (0.90 mmol) of lead tetra­acetate were added. The resulting mixture was stirred at 353 K for 24 h. The solvents were removed under reduced pressure (Fig. 5 ▸). The product was isolated by column chromatography (eluent AcOEt:CDCl_3_ = 0.5:9.5; *R*_f_ 0.6). A single crystal of compound (**4**) was obtained by slow evaporation of a chloro­form solution. (**4**): Yield 77 mg (30%). ^1^H NMR (300 MHz, DMSO-*d*_6_): *δ =* 2.34 (*s*, 3H, OAc), 2.43 (*s*, 3H, OAc), 2.48 (*s*, 3H, CH_3_), 3.87 (*s*, 3H, OCH_3_), 6.87 (*s*, 1H, H-5 (benzo­thia­zole), 7.38–7.41 (*m*, 2H, C_6_H_4_CH_3_), 7.45–7.52 (*m*, 3H, Ph), 7.69–7.77 (*m*, 3H, Ph+NH), 8.48–8.52 (*m*, C_6_H_4_CH_3_), 9.99 (*br. s*, 1H, NH). Mass-spectrum, *m*/*z* (*I_r_*_el_, %): *m*/*z*: 569.15 [M+H]^+^ (100). Found, %: C 61.28, H 4.24, N 14.77. C_29_H_24_N_6_O_5_S. Calculated, %: C 61.26, H 4.25, N 14.78.

## 7. Refinement

Crystal data, data collection and structure refinement details are summarized in Table 2 ▸. The H atoms of the NH_2_ groups were found in difference-Fourier maps and were refined with *U*_iso_(H) = 1.2*U*_eq_(N). The C-bound H atoms were positioned geometrically (C—H = 0.93–0.96 Å) and refined using a riding model, with *U*_iso_(H) = 1.2 or 1.5*U*_eq_(C). The oxygen atom (O5) of the carbonyl group exhibits disorder at two positions with a final occupancy ratio of 0.617 (4):0.383 (4). The disordered atom was modelled using SADI and EADP constraints.

## Supplementary Material

Crystal structure: contains datablock(s) I. DOI: 10.1107/S2056989026008972/ee2032sup1.cif

Structure factors: contains datablock(s) I. DOI: 10.1107/S2056989026008972/ee2032Isup2.hkl

Supporting information file. DOI: 10.1107/S2056989026008972/ee2032Isup3.cml

CCDC reference: 2584107

Additional supporting information:  crystallographic information; 3D view; checkCIF report

## Figures and Tables

**Table 1:** Hydrogen-bond geometry (Å, °)

*D*—H⋯*A*	*D*—H	H⋯*A*	*D*⋯*A*	*D*—H⋯*A*
N3—H3*A*⋯O2^i^	0.87 (2)	2.59 (2)	3.229 (3)	130.5 (18)
N3—H3*A*⋯N4	0.87 (2)	2.23 (2)	2.613 (2)	106.8 (17)
N3—H3*B*⋯N1	0.93 (2)	1.97 (2)	2.676 (2)	130.8 (19)
C17—H17⋯O5^ii^	0.93	2.55	3.390 (5)	150
C26—H26*B*⋯O2^iii^	0.96	2.58	3.390 (4)	143
C27—H27*A*⋯O3^iv^	0.96	2.53	3.389 (4)	150
C27—H27*B*⋯O5*A*^v^	0.96	2.29	3.194 (8)	156

Symmetry codes: (i) 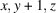; (ii) 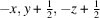; (iii) 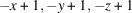; (iv) 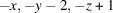; (v) 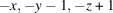.

**Table 2:** Experimental details

Crystal data	
Chemical formula	C_29_H_24_N_6_O_5_S
*M*_r_	568.60
Crystal system, space group	Monoclinic, *P*2_1_/*c*
Temperature (K)	293
*a*, *b*, *c* (Å)	19.7610 (5), 5.8264 (2), 24.3643 (5)
β (°)	94.711 (2)
*V* (Å^3^)	2795.72 (13)
*Z*	4
Radiation type	Mo *K*α
μ (mm^−1^)	0.17
Crystal size (mm)	0.41 × 0.25 × 0.16

Data collection	
Diffractometer	XtaLAB Synergy, Dualflex, HyPix
Absorption correction	Multi-scan (*CrysAlis PRO*; Rigaku OD, 2021 ▸)
*T*_min_, *T*_max_	0.951, 0.974
No. of measured, independent and observed [*I* > 2σ(*I*)] reflections	94505, 7321, 3876
*R*_int_	0.094
(sin θ/λ)_max_ (Å^−1^)	0.695

Refinement	
*R*[*F*^2^ > 2σ(*F*^2^)], *wR*(*F*^2^), *S*	0.052, 0.150, 1.04
No. of reflections	7321
No. of parameters	384
No. of restraints	1
H-atom treatment	H atoms treated by a mixture of independent and constrained refinement
Δρ_max_, Δρ_min_ (e Å^−3^)	0.21, −0.20

Computer programs: *CrysAlis PRO* (Rigaku OD, 2021 ▸), *SHELXT* (Sheldrick, 2015*a* ▸), *SHELXL* (Sheldrick, 2015*b* ▸), *ORTEP-3 for Windows* (Farrugia, 2012 ▸) and *PLATON* (Spek, 2020 ▸).

## supplementary crystallographic information

### (Z)-2-({Amino[6-phenyl-3-(4-methylphenyl)-1,2,4-triazin-5-yl]methylidene}amino)-6-methoxybenzo[d]thiazole-4,7-diyl diacetate Crystal data

**Table:**

C_29_H_24_N_6_O_5_S	*F*(000) = 1184
*M**_r_* = 568.60	*D*_x_ = 1.351 Mg m^−^^3^
Monoclinic, *P*2_1_/*c*	Mo *K*α radiation, λ = 0.71073 Å
*a* = 19.7610 (5) Å	Cell parameters from 14058 reflections
*b* = 5.8264 (2) Å	θ = 1.7–22.1°
*c* = 24.3643 (5) Å	µ = 0.17 mm^−^^1^
β = 94.711 (2)°	*T* = 293 K
*V* = 2795.72 (13) Å^3^	Prism, red
*Z* = 4	0.41 × 0.25 × 0.16 mm

### (Z)-2-({Amino[6-phenyl-3-(4-methylphenyl)-1,2,4-triazin-5-yl]methylidene}amino)-6-methoxybenzo[d]thiazole-4,7-diyl diacetate Data collection

**Table:**

XtaLAB Synergy, Dualflex, HyPix diffractometer	7321 independent reflections
Radiation source: micro-focus sealed X-ray tube, PhotonJet (Mo) X-ray Source	3876 reflections with *I* > 2σ(*I*)
Mirror monochromator	*R*_int_ = 0.094
Detector resolution: 10.0000 pixels mm^-1^	θ_max_ = 29.6°, θ_min_ = 1.7°
ω scans	*h* = −26→27
Absorption correction: multi-scan (CrysAlisPro; Rigaku OD, 2021)	*k* = −8→8
*T*_min_ = 0.951, *T*_max_ = 0.974	*l* = −33→32
94505 measured reflections	

### (Z)-2-({Amino[6-phenyl-3-(4-methylphenyl)-1,2,4-triazin-5-yl]methylidene}amino)-6-methoxybenzo[d]thiazole-4,7-diyl diacetate Refinement

**Table:**

Refinement on *F*^2^	Primary atom site location: dual
Least-squares matrix: full	Hydrogen site location: mixed
*R*[*F*^2^ > 2σ(*F*^2^)] = 0.052	H atoms treated by a mixture of independent and constrained refinement
*wR*(*F*^2^) = 0.150	*w* = 1/[σ^2^(*F*_o_^2^) + (0.0629*P*)^2^ + 0.2039*P*] where *P* = (*F*_o_^2^ + 2*F*_c_^2^)/3
*S* = 1.04	(Δ/σ)_max_ = 0.001
7321 reflections	Δρ_max_ = 0.21 e Å^−^^3^
384 parameters	Δρ_min_ = −0.20 e Å^−^^3^
1 restraint	

### (Z)-2-({Amino[6-phenyl-3-(4-methylphenyl)-1,2,4-triazin-5-yl]methylidene}amino)-6-methoxybenzo[d]thiazole-4,7-diyl diacetate Special details

**Table:**

Geometry. All esds (except the esd in the dihedral angle between two l.s. planes) are estimated using the full covariance matrix. The cell esds are taken into account individually in the estimation of esds in distances, angles and torsion angles; correlations between esds in cell parameters are only used when they are defined by crystal symmetry. An approximate (isotropic) treatment of cell esds is used for estimating esds involving l.s. planes.

### (Z)-2-({Amino[6-phenyl-3-(4-methylphenyl)-1,2,4-triazin-5-yl]methylidene}amino)-6-methoxybenzo[d]thiazole-4,7-diyl diacetate Fractional atomic coordinates and isotropic or equivalent isotropic displacement parameters (Å^2^)

**Table:**

	*x*	*y*	*z*	*U*_iso_*/*U*_eq_	Occ. (<1)
C1	0.21371 (10)	−0.0169 (3)	0.37633 (8)	0.0501 (5)	
C2	0.17864 (10)	−0.2051 (3)	0.44712 (8)	0.0513 (5)	
C3	0.14532 (10)	−0.3400 (3)	0.40576 (8)	0.0506 (5)	
C4	0.10892 (10)	−0.5327 (3)	0.41903 (8)	0.0549 (5)	
C5	0.10454 (11)	−0.5896 (4)	0.47403 (9)	0.0634 (6)	
C6	0.13858 (12)	−0.4578 (4)	0.51514 (9)	0.0684 (6)	
H6	0.136281	−0.497031	0.551938	0.082*	
C7	0.17540 (11)	−0.2705 (4)	0.50151 (8)	0.0599 (5)	
C8	0.27976 (17)	−0.1373 (5)	0.54359 (10)	0.0828 (8)	
C9	0.27880 (10)	0.3049 (3)	0.36523 (8)	0.0482 (5)	
C10	0.31600 (10)	0.4545 (3)	0.32773 (7)	0.0486 (5)	
C11	0.39337 (10)	0.7418 (4)	0.32262 (8)	0.0526 (5)	
C12	0.31716 (10)	0.4223 (4)	0.27052 (8)	0.0536 (5)	
C13	0.27288 (12)	0.2688 (4)	0.23534 (8)	0.0579 (5)	
C14	0.30056 (15)	0.0875 (4)	0.20888 (10)	0.0819 (7)	
H14	0.347286	0.064348	0.212072	0.098*	
C15	0.2585 (2)	−0.0606 (5)	0.17743 (12)	0.1074 (11)	
H15	0.277110	−0.187109	0.160910	0.129*	
C16	0.1911 (2)	−0.0243 (6)	0.17039 (11)	0.1051 (10)	
H16	0.163484	−0.124743	0.149010	0.126*	
C17	0.16345 (16)	0.1595 (6)	0.19459 (11)	0.0970 (9)	
H17	0.117027	0.186958	0.189062	0.116*	
C18	0.20404 (13)	0.3049 (5)	0.22724 (10)	0.0787 (7)	
H18	0.184695	0.429212	0.244069	0.094*	
C19	0.30912 (18)	0.0520 (6)	0.57940 (12)	0.1174 (11)	
H19A	0.306157	0.193856	0.559294	0.176*	
H19B	0.355842	0.018822	0.590444	0.176*	
H19C	0.284211	0.065047	0.611465	0.176*	
C20	0.43176 (10)	0.9356 (4)	0.34862 (8)	0.0545 (5)	
C21	0.42356 (11)	0.9989 (4)	0.40250 (9)	0.0660 (6)	
H21	0.393252	0.918715	0.422600	0.079*	
C22	0.45978 (13)	1.1791 (4)	0.42650 (10)	0.0745 (7)	
H22	0.453821	1.217824	0.462820	0.089*	
C23	0.50483 (11)	1.3039 (4)	0.39798 (10)	0.0690 (6)	
C24	0.51209 (12)	1.2416 (4)	0.34442 (10)	0.0771 (7)	
H24	0.541801	1.323756	0.324156	0.093*	
C25	0.47656 (12)	1.0611 (4)	0.32009 (9)	0.0716 (6)	
H25	0.482770	1.022793	0.283793	0.086*	
C26	0.54425 (14)	1.5043 (5)	0.42390 (12)	0.0943 (8)	
H26A	0.571666	1.571426	0.397380	0.141*	
H26B	0.572946	1.451480	0.455052	0.141*	
H26C	0.513155	1.617094	0.435718	0.141*	
C27	0.06120 (16)	−0.8397 (5)	0.54036 (11)	0.0985 (9)	
H27A	0.036967	−0.981971	0.542078	0.148*	
H27B	0.036742	−0.720809	0.557540	0.148*	
H27C	0.105512	−0.855784	0.559273	0.148*	
C28	0.02414 (14)	−0.6465 (6)	0.35039 (12)	0.0957 (9)	
C29	0.00301 (16)	−0.8326 (5)	0.31195 (13)	0.1107 (11)	
H29A	0.036148	−0.850600	0.285579	0.166*	
H29B	−0.040186	−0.795503	0.293116	0.166*	
H29C	−0.000710	−0.973075	0.332058	0.166*	
N1	0.21598 (8)	−0.0226 (3)	0.42984 (6)	0.0517 (4)	
N2	0.24549 (8)	0.1299 (3)	0.34327 (6)	0.0532 (4)	
N3	0.28487 (9)	0.3671 (3)	0.41783 (7)	0.0552 (4)	
H3A	0.3075 (11)	0.491 (4)	0.4276 (9)	0.066*	
H3B	0.2614 (11)	0.277 (4)	0.4415 (9)	0.066*	
N4	0.35295 (8)	0.6197 (3)	0.35291 (6)	0.0510 (4)	
N5	0.40296 (10)	0.6934 (3)	0.27026 (7)	0.0698 (5)	
N6	0.36328 (10)	0.5355 (3)	0.24410 (7)	0.0698 (5)	
O1	0.20970 (9)	−0.1371 (3)	0.54256 (6)	0.0756 (5)	
O2	0.31015 (11)	−0.2704 (4)	0.51763 (9)	0.1052 (7)	
O3	0.06773 (9)	−0.7806 (3)	0.48390 (7)	0.0806 (5)	
O4	0.08296 (7)	−0.6852 (3)	0.37831 (6)	0.0671 (4)	
O5	0.0017 (2)	−0.4394 (7)	0.34761 (19)	0.1130 (14)	0.617 (4)
O5A	−0.0205 (3)	−0.5585 (12)	0.3785 (3)	0.1130 (14)	0.383 (4)
S1	0.16294 (3)	−0.23292 (9)	0.34217 (2)	0.05618 (17)	

### (Z)-2-({Amino[6-phenyl-3-(4-methylphenyl)-1,2,4-triazin-5-yl]methylidene}amino)-6-methoxybenzo[d]thiazole-4,7-diyl diacetate Atomic displacement parameters (Å^2^)

**Table:**

	*U*^11^	*U*^22^	*U*^33^	*U*^12^	*U*^13^	*U*^23^
C1	0.0507 (11)	0.0490 (11)	0.0500 (11)	0.0021 (9)	0.0011 (9)	−0.0068 (9)
C2	0.0538 (12)	0.0486 (11)	0.0514 (11)	−0.0045 (9)	0.0048 (9)	−0.0061 (9)
C3	0.0480 (11)	0.0484 (11)	0.0546 (11)	0.0008 (9)	−0.0008 (9)	−0.0062 (9)
C4	0.0505 (11)	0.0480 (12)	0.0650 (13)	−0.0035 (9)	−0.0036 (9)	−0.0115 (10)
C5	0.0621 (13)	0.0540 (13)	0.0746 (15)	−0.0123 (11)	0.0078 (11)	−0.0011 (11)
C6	0.0837 (16)	0.0635 (15)	0.0586 (13)	−0.0206 (12)	0.0094 (11)	−0.0043 (11)
C7	0.0716 (14)	0.0574 (13)	0.0507 (12)	−0.0142 (11)	0.0054 (10)	−0.0086 (10)
C8	0.106 (2)	0.084 (2)	0.0546 (14)	−0.0285 (17)	−0.0156 (14)	0.0050 (13)
C9	0.0467 (11)	0.0510 (12)	0.0470 (11)	0.0048 (9)	0.0034 (8)	−0.0007 (9)
C10	0.0474 (11)	0.0502 (11)	0.0482 (11)	0.0054 (9)	0.0049 (9)	−0.0010 (9)
C11	0.0492 (11)	0.0595 (13)	0.0497 (11)	−0.0002 (10)	0.0078 (9)	0.0014 (9)
C12	0.0573 (12)	0.0552 (12)	0.0484 (11)	0.0045 (10)	0.0060 (9)	−0.0005 (9)
C13	0.0744 (15)	0.0578 (13)	0.0412 (10)	−0.0001 (11)	0.0042 (10)	−0.0011 (9)
C14	0.109 (2)	0.0693 (17)	0.0663 (14)	0.0119 (15)	0.0009 (14)	−0.0127 (13)
C15	0.166 (3)	0.074 (2)	0.0796 (19)	0.007 (2)	−0.005 (2)	−0.0281 (15)
C16	0.153 (3)	0.090 (2)	0.0660 (17)	−0.030 (2)	−0.0258 (19)	−0.0088 (16)
C17	0.091 (2)	0.119 (3)	0.0759 (17)	−0.0212 (18)	−0.0224 (15)	−0.0093 (18)
C18	0.0767 (17)	0.0887 (19)	0.0687 (15)	−0.0005 (14)	−0.0063 (13)	−0.0146 (13)
C19	0.152 (3)	0.104 (2)	0.0905 (19)	−0.059 (2)	−0.0240 (18)	−0.0082 (17)
C20	0.0479 (11)	0.0624 (13)	0.0533 (12)	−0.0021 (10)	0.0048 (9)	0.0010 (10)
C21	0.0655 (14)	0.0714 (16)	0.0623 (13)	−0.0108 (12)	0.0122 (11)	−0.0041 (11)
C22	0.0804 (16)	0.0771 (17)	0.0664 (14)	−0.0115 (14)	0.0088 (12)	−0.0146 (12)
C23	0.0580 (13)	0.0686 (15)	0.0793 (16)	−0.0048 (11)	−0.0018 (12)	−0.0066 (12)
C24	0.0677 (15)	0.0888 (19)	0.0762 (16)	−0.0219 (14)	0.0147 (12)	−0.0011 (14)
C25	0.0703 (15)	0.0850 (17)	0.0605 (13)	−0.0202 (13)	0.0115 (11)	−0.0060 (12)
C26	0.0827 (18)	0.086 (2)	0.112 (2)	−0.0183 (15)	−0.0043 (15)	−0.0200 (16)
C27	0.114 (2)	0.087 (2)	0.096 (2)	−0.0385 (18)	0.0131 (17)	0.0159 (16)
C28	0.0722 (17)	0.100 (2)	0.109 (2)	0.0191 (16)	−0.0275 (15)	−0.0398 (18)
C29	0.099 (2)	0.100 (2)	0.126 (2)	−0.0037 (18)	−0.0388 (18)	−0.0402 (19)
N1	0.0569 (10)	0.0491 (10)	0.0489 (9)	−0.0057 (8)	0.0031 (7)	−0.0051 (7)
N2	0.0586 (10)	0.0508 (10)	0.0500 (9)	−0.0047 (8)	0.0038 (8)	−0.0033 (8)
N3	0.0621 (11)	0.0569 (11)	0.0469 (10)	−0.0117 (9)	0.0064 (8)	−0.0034 (8)
N4	0.0518 (9)	0.0542 (10)	0.0474 (9)	−0.0014 (8)	0.0066 (7)	−0.0017 (8)
N5	0.0754 (13)	0.0807 (14)	0.0555 (11)	−0.0163 (11)	0.0188 (10)	−0.0080 (10)
N6	0.0803 (13)	0.0765 (13)	0.0540 (10)	−0.0130 (11)	0.0133 (10)	−0.0072 (9)
O1	0.1031 (13)	0.0737 (11)	0.0502 (8)	−0.0307 (10)	0.0067 (8)	−0.0101 (7)
O2	0.0929 (14)	0.1154 (17)	0.1015 (15)	−0.0004 (12)	−0.0275 (12)	−0.0177 (13)
O3	0.0867 (12)	0.0673 (11)	0.0879 (12)	−0.0310 (9)	0.0085 (9)	0.0015 (9)
O4	0.0606 (9)	0.0546 (9)	0.0831 (10)	−0.0025 (7)	−0.0129 (8)	−0.0165 (7)
O5	0.092 (2)	0.085 (3)	0.152 (4)	0.026 (2)	−0.051 (2)	−0.031 (2)
O5A	0.092 (2)	0.085 (3)	0.152 (4)	0.026 (2)	−0.051 (2)	−0.031 (2)
S1	0.0613 (3)	0.0532 (3)	0.0525 (3)	−0.0023 (2)	−0.0044 (2)	−0.0077 (2)

### (Z)-2-({Amino[6-phenyl-3-(4-methylphenyl)-1,2,4-triazin-5-yl]methylidene}amino)-6-methoxybenzo[d]thiazole-4,7-diyl diacetate Geometric parameters (Å, º)

**Table:**

C1—N1	1.301 (2)	C16—H16	0.9300
C1—N2	1.363 (2)	C17—C18	1.374 (4)
C1—S1	1.774 (2)	C17—H17	0.9300
C2—N1	1.380 (2)	C18—H18	0.9300
C2—C7	1.385 (3)	C19—H19A	0.9600
C2—C3	1.399 (3)	C19—H19B	0.9600
C3—C4	1.386 (3)	C19—H19C	0.9600
C3—S1	1.732 (2)	C20—C25	1.379 (3)
C4—C5	1.390 (3)	C20—C21	1.386 (3)
C4—O4	1.398 (2)	C21—C22	1.374 (3)
C5—O3	1.362 (2)	C21—H21	0.9300
C5—C6	1.391 (3)	C22—C23	1.381 (3)
C6—C7	1.368 (3)	C22—H22	0.9300
C6—H6	0.9300	C23—C24	1.373 (3)
C7—O1	1.397 (2)	C23—C26	1.512 (3)
C8—O2	1.193 (3)	C24—C25	1.372 (3)
C8—O1	1.383 (3)	C24—H24	0.9300
C8—C19	1.494 (4)	C25—H25	0.9300
C9—N2	1.305 (2)	C26—H26A	0.9600
C9—N3	1.328 (2)	C26—H26B	0.9600
C9—C10	1.498 (3)	C26—H26C	0.9600
C10—N4	1.328 (2)	C27—O3	1.434 (3)
C10—C12	1.408 (3)	C27—H27A	0.9600
C11—N5	1.335 (2)	C27—H27B	0.9600
C11—N4	1.336 (2)	C27—H27C	0.9600
C11—C20	1.474 (3)	C28—O5A	1.269 (7)
C12—N6	1.333 (3)	C28—O5	1.285 (4)
C12—C13	1.475 (3)	C28—O4	1.317 (3)
C13—C14	1.374 (3)	C28—C29	1.471 (4)
C13—C18	1.375 (3)	C29—H29A	0.9600
C14—C15	1.385 (4)	C29—H29B	0.9600
C14—H14	0.9300	C29—H29C	0.9600
C15—C16	1.346 (4)	N3—H3A	0.87 (2)
C15—H15	0.9300	N3—H3B	0.93 (2)
C16—C17	1.358 (4)	N5—N6	1.336 (3)
			
N1—C1—N2	128.96 (18)	C8—C19—H19C	109.5
N1—C1—S1	115.04 (15)	H19A—C19—H19C	109.5
N2—C1—S1	115.99 (14)	H19B—C19—H19C	109.5
N1—C2—C7	124.86 (17)	C25—C20—C21	117.8 (2)
N1—C2—C3	116.42 (17)	C25—C20—C11	121.24 (18)
C7—C2—C3	118.60 (18)	C21—C20—C11	120.99 (18)
C4—C3—C2	120.64 (18)	C22—C21—C20	120.6 (2)
C4—C3—S1	130.31 (16)	C22—C21—H21	119.7
C2—C3—S1	108.94 (15)	C20—C21—H21	119.7
C3—C4—C5	119.52 (18)	C21—C22—C23	121.6 (2)
C3—C4—O4	121.12 (18)	C21—C22—H22	119.2
C5—C4—O4	118.96 (18)	C23—C22—H22	119.2
O3—C5—C4	116.21 (19)	C24—C23—C22	117.4 (2)
O3—C5—C6	123.9 (2)	C24—C23—C26	120.7 (2)
C4—C5—C6	119.85 (19)	C22—C23—C26	121.9 (2)
C7—C6—C5	120.1 (2)	C25—C24—C23	121.6 (2)
C7—C6—H6	120.0	C25—C24—H24	119.2
C5—C6—H6	120.0	C23—C24—H24	119.2
C6—C7—C2	121.26 (19)	C24—C25—C20	121.0 (2)
C6—C7—O1	120.43 (18)	C24—C25—H25	119.5
C2—C7—O1	118.29 (18)	C20—C25—H25	119.5
O2—C8—O1	122.5 (2)	C23—C26—H26A	109.5
O2—C8—C19	127.0 (3)	C23—C26—H26B	109.5
O1—C8—C19	110.5 (3)	H26A—C26—H26B	109.5
N2—C9—N3	127.71 (18)	C23—C26—H26C	109.5
N2—C9—C10	117.43 (16)	H26A—C26—H26C	109.5
N3—C9—C10	114.85 (18)	H26B—C26—H26C	109.5
N4—C10—C12	120.05 (17)	O3—C27—H27A	109.5
N4—C10—C9	114.74 (16)	O3—C27—H27B	109.5
C12—C10—C9	125.09 (18)	H27A—C27—H27B	109.5
N5—C11—N4	123.62 (19)	O3—C27—H27C	109.5
N5—C11—C20	117.64 (17)	H27A—C27—H27C	109.5
N4—C11—C20	118.70 (17)	H27B—C27—H27C	109.5
N6—C12—C10	118.65 (19)	O5A—C28—O4	114.4 (4)
N6—C12—C13	114.57 (17)	O5—C28—O4	118.3 (3)
C10—C12—C13	126.76 (18)	O5A—C28—C29	117.9 (4)
C14—C13—C18	118.5 (2)	O5—C28—C29	125.5 (3)
C14—C13—C12	119.9 (2)	O4—C28—C29	113.3 (2)
C18—C13—C12	121.6 (2)	C28—C29—H29A	109.5
C13—C14—C15	119.6 (3)	C28—C29—H29B	109.5
C13—C14—H14	120.2	H29A—C29—H29B	109.5
C15—C14—H14	120.2	C28—C29—H29C	109.5
C16—C15—C14	121.0 (3)	H29A—C29—H29C	109.5
C16—C15—H15	119.5	H29B—C29—H29C	109.5
C14—C15—H15	119.5	C1—N1—C2	110.51 (16)
C15—C16—C17	119.9 (3)	C9—N2—C1	119.31 (16)
C15—C16—H16	120.1	C9—N3—H3A	119.6 (14)
C17—C16—H16	120.1	C9—N3—H3B	115.7 (13)
C16—C17—C18	120.0 (3)	H3A—N3—H3B	124.6 (19)
C16—C17—H17	120.0	C10—N4—C11	117.49 (16)
C18—C17—H17	120.0	C11—N5—N6	118.52 (17)
C17—C18—C13	120.8 (3)	C12—N6—N5	120.54 (17)
C17—C18—H18	119.6	C8—O1—C7	116.00 (18)
C13—C18—H18	119.6	C5—O3—C27	117.19 (18)
C8—C19—H19A	109.5	C28—O4—C4	120.97 (18)
C8—C19—H19B	109.5	C3—S1—C1	89.06 (9)
H19A—C19—H19B	109.5		
			
N1—C2—C3—C4	177.28 (18)	N4—C11—C20—C21	−3.5 (3)
C7—C2—C3—C4	1.1 (3)	C25—C20—C21—C22	−0.9 (3)
N1—C2—C3—S1	0.8 (2)	C11—C20—C21—C22	179.7 (2)
C7—C2—C3—S1	−175.45 (16)	C20—C21—C22—C23	0.6 (4)
C2—C3—C4—C5	1.2 (3)	C21—C22—C23—C24	0.1 (4)
S1—C3—C4—C5	176.86 (17)	C21—C22—C23—C26	179.2 (2)
C2—C3—C4—O4	−171.53 (18)	C22—C23—C24—C25	−0.6 (4)
S1—C3—C4—O4	4.2 (3)	C26—C23—C24—C25	−179.7 (2)
C3—C4—C5—O3	179.76 (19)	C23—C24—C25—C20	0.3 (4)
O4—C4—C5—O3	−7.4 (3)	C21—C20—C25—C24	0.4 (4)
C3—C4—C5—C6	−2.2 (3)	C11—C20—C25—C24	179.9 (2)
O4—C4—C5—C6	170.6 (2)	N2—C1—N1—C2	−177.39 (19)
O3—C5—C6—C7	178.9 (2)	S1—C1—N1—C2	1.7 (2)
C4—C5—C6—C7	1.0 (4)	C7—C2—N1—C1	174.3 (2)
C5—C6—C7—C2	1.3 (4)	C3—C2—N1—C1	−1.6 (2)
C5—C6—C7—O1	180.0 (2)	N3—C9—N2—C1	−2.1 (3)
N1—C2—C7—C6	−178.2 (2)	C10—C9—N2—C1	176.82 (17)
C3—C2—C7—C6	−2.3 (3)	N1—C1—N2—C9	−6.8 (3)
N1—C2—C7—O1	3.1 (3)	S1—C1—N2—C9	174.04 (14)
C3—C2—C7—O1	178.99 (18)	C12—C10—N4—C11	−3.8 (3)
N2—C9—C10—N4	−175.32 (17)	C9—C10—N4—C11	172.47 (16)
N3—C9—C10—N4	3.8 (2)	N5—C11—N4—C10	−6.2 (3)
N2—C9—C10—C12	0.8 (3)	C20—C11—N4—C10	176.25 (17)
N3—C9—C10—C12	179.85 (18)	N4—C11—N5—N6	9.7 (3)
N4—C10—C12—N6	10.2 (3)	C20—C11—N5—N6	−172.69 (19)
C9—C10—C12—N6	−165.74 (19)	C10—C12—N6—N5	−6.7 (3)
N4—C10—C12—C13	−171.40 (19)	C13—C12—N6—N5	174.70 (19)
C9—C10—C12—C13	12.7 (3)	C11—N5—N6—C12	−2.8 (3)
N6—C12—C13—C14	62.2 (3)	O2—C8—O1—C7	−12.1 (4)
C10—C12—C13—C14	−116.3 (2)	C19—C8—O1—C7	166.2 (2)
N6—C12—C13—C18	−116.5 (2)	C6—C7—O1—C8	114.8 (2)
C10—C12—C13—C18	65.1 (3)	C2—C7—O1—C8	−66.5 (3)
C18—C13—C14—C15	−3.5 (4)	C4—C5—O3—C27	−178.6 (2)
C12—C13—C14—C15	177.8 (2)	C6—C5—O3—C27	3.5 (4)
C13—C14—C15—C16	2.9 (5)	O5A—C28—O4—C4	−37.2 (6)
C14—C15—C16—C17	−0.3 (5)	O5—C28—O4—C4	22.3 (5)
C15—C16—C17—C18	−1.5 (5)	C29—C28—O4—C4	−176.2 (2)
C16—C17—C18—C13	0.8 (4)	C3—C4—O4—C28	−84.3 (3)
C14—C13—C18—C17	1.7 (4)	C5—C4—O4—C28	102.9 (3)
C12—C13—C18—C17	−179.6 (2)	C4—C3—S1—C1	−175.9 (2)
N5—C11—C20—C25	−0.7 (3)	C2—C3—S1—C1	0.18 (15)
N4—C11—C20—C25	177.1 (2)	N1—C1—S1—C3	−1.14 (16)
N5—C11—C20—C21	178.8 (2)	N2—C1—S1—C3	178.10 (16)

### (Z)-2-({Amino[6-phenyl-3-(4-methylphenyl)-1,2,4-triazin-5-yl]methylidene}amino)-6-methoxybenzo[d]thiazole-4,7-diyl diacetate Hydrogen-bond geometry (Å, º)

**Table:**

*D*—H···*A*	*D*—H	H···*A*	*D*···*A*	*D*—H···*A*
N3—H3*A*···O2^i^	0.87 (2)	2.59 (2)	3.229 (3)	130.5 (18)
N3—H3*A*···N4	0.87 (2)	2.23 (2)	2.613 (2)	106.8 (17)
N3—H3*B*···N1	0.93 (2)	1.97 (2)	2.676 (2)	130.8 (19)
C17—H17···O5^ii^	0.93	2.55	3.390 (5)	150
C21—H21···N4	0.93	2.52	2.830 (3)	100
C25—H25···N5	0.93	2.49	2.809 (3)	100
C26—H26*B*···O2^iii^	0.96	2.58	3.390 (4)	143
C27—H27*A*···O3^iv^	0.96	2.53	3.389 (4)	150
C27—H27*B*···O5*A*^v^	0.96	2.29	3.194 (8)	156

Symmetry codes: (i) *x*, *y*+1, *z*; (ii) −*x*, *y*+1/2, −*z*+1/2; (iii) −*x*+1, −*y*+1, −*z*+1; (iv) −*x*, −*y*−2, −*z*+1; (v) −*x*, −*y*−1, −*z*+1.
